# Incorporation of natural and synthetic polymers into honey hydrogel for wound healing: A review

**DOI:** 10.1002/hsr2.2251

**Published:** 2024-07-16

**Authors:** Siau Wui Chin, Adzzie‐Shazleen Azman, Ji Wei Tan

**Affiliations:** ^1^ School of Science Monash University Malaysia Subang Jaya Malaysia

**Keywords:** honey hydrogel, natural and synthetic polymers, wound dressings, wound healing

## Abstract

**Background and Aims:**

The difficulty in treating chronic wounds due to the prolonged inflammation stage has affected a staggering 6.5 million people, accompanied by 25 billion USD annually in the United States alone. A 1.9% rise in chronic wound prevalence among Medicare beneficiaries was reported from 2014 to 2019. Besides, the global wound care market values were anticipated to increase from USD 20.18 billion in 2022 to USD 30.52 billion in 2030, suggesting an expected rise in chronic wounds financial burdens. The lack of feasibility in using traditional dry wound dressings sparks hydrogel development as an alternative approach to tackling chronic wounds. Since ancient times, honey has been used to treat wounds, including burns, and ongoing studies have also demonstrated its wound‐healing capabilities on cellular and animal models. However, the fluidity and low mechanical strength in honey hydrogel necessitate the incorporation of other polymers. Therefore, this review aims to unravel the characteristics and feasibility of natural (chitosan and gelatin) and synthetic (polyvinyl alcohol and polyethylene glycol) polymers to be incorporated in the honey hydrogel.

**Methods:**

Relevant articles were identified from databases (PubMed, Google Scholar, and Science Direct) using keywords related to honey, hydrogel, and polymers. Relevant data from selected studies were synthesized narratively and reported following a structured narrative format.

**Results:**

The importance of honey's roles and mechanisms of action in wound dressings were discussed. Notable studies concerning honey hydrogels with diverse polymers were also included in this article to provide a better perspective on fabricating customized hydrogel wound dressings for various types of wounds in the future.

**Conclusion:**

Honey's incapability to stand alone in hydrogel requires the incorporation of natural and synthetic polymers into the hydrogel. With this review, it is hoped that the fabrication and commercialization of the desired honey composite hydrogel for wound treatment could be brought forth.

## Key points

- The inappropriate use of traditional wound dressings sparks the development of hydrogel as an alternative approach to tackle wounds that are hard to heal.
- The honey hydrogel has been demonstrated to contain wound healing capabilities. However, due to its liquid nature, honey alone is unsuitable for creating hydrogel scaffolds, leading to its low therapeutic properties on wounds.
- Incorporation of natural and synthetic polymers to produce a honey composite hydrogel with better mechanical strength and an increase in the honey's wound healing properties. Thus, it may be used as a potential dressing for different types of wounds in the future.

## 1 INTRODUCTION

To date, chronic wounds do not heal at the stipulated time frame and often take years to recover. In particular, chronic wounds were the result of venous insufficiency, diabetes, neuropathies, and immune dysfunction complications.^1^ Chronic wounds have affected over 6.5 million people in the United States alone, with a global prevalence range of 1.51−2.21 per 1000 people.^2^ A 2014−2019 retrospective analysis suggested a rise in chronic wound prevalence from 14.5% to 16.4%, increasing the number of Medicare beneficiaries from 8.2 million to 10.5 million.^3^ Chronic wounds have accounted for over 25 billion USD per year.^4^ The financial burden is expected to increase by 4.61% compound annual growth rate, increasing the Global Wound Care market value from USD 20.18 billion (the year 2022) to USD 30.52 billion (the year 2030).^5^ To no doubt, the high recurrence rate of diabetic foot ulcers at 66% adds difficulty in managing chronic wounds.^6^ Atop the economic burden, chronic wounds limit mobility, causing severe emotional and physical distress to the patients.^7^ If left untreated, infected wounds may potentially necessitate amputation or even result in death.^7^

Conventional wound dressings such as bandages and gauzes are undesired for chronic wound applications as they are dry and require regular dressing changes to prevent healthy tissue maceration.^8^ These have led to the revolution of modern wet wound dressings, such as hydrogels, foams, films, and hydrocolloids. Among these, hydrogels, a three‐dimensional gel‐like material, stand out for their superior biodegradability, high absorbance capacity, and versatility to meet various requirements.^9^

Honey was revisited for wound healing after the increased emergence of antibiotic resistance, which accounted for approximately 1.27 million deaths worldwide.^10^ Notably, honey possesses a broad spectrum of antimicrobial activities and has not been documented for microbial resistance.^11^, ^12^ Honey serves multiple purposes in the hydrogel, including reducing inflammation and infection risk and promoting tissue regeneration and angiogenesis.^13^ Honey's high viscosity contributes to the hydrogel's elasticity and adhesiveness.^13^ However, honey alone cannot create a solid hydrogel wound dressing due to its fluidity and low mechanical strength. Thus, natural or synthetic polymers should be incorporated to fabricate a feasible honey hydrogel dressing for various wounds.

This review sheds light on the possible polymers that could fabricate a honey hydrogel wound dressing. As such, the significant roles of polymers such as chitosan (CS), gelatin, polyvinyl alcohol (PVA), and polyethylene glycol (PEG) will be further discussed.

## 2 LITERATURE SEARCH

Relevant articles were identified from databases (PubMed, Google Scholar, and Science Direct) using keywords: wound healing, traditional and modern wound dressings, benefits of honey in wound healing, hydrogel wound dressings, and natural and synthetic polymers for hydrogel wound dressings. Filter was applied to include authenticated research articles in English and data published from 2010 to 2023.

## 3 WOUND HEALING

**Figure 1:**
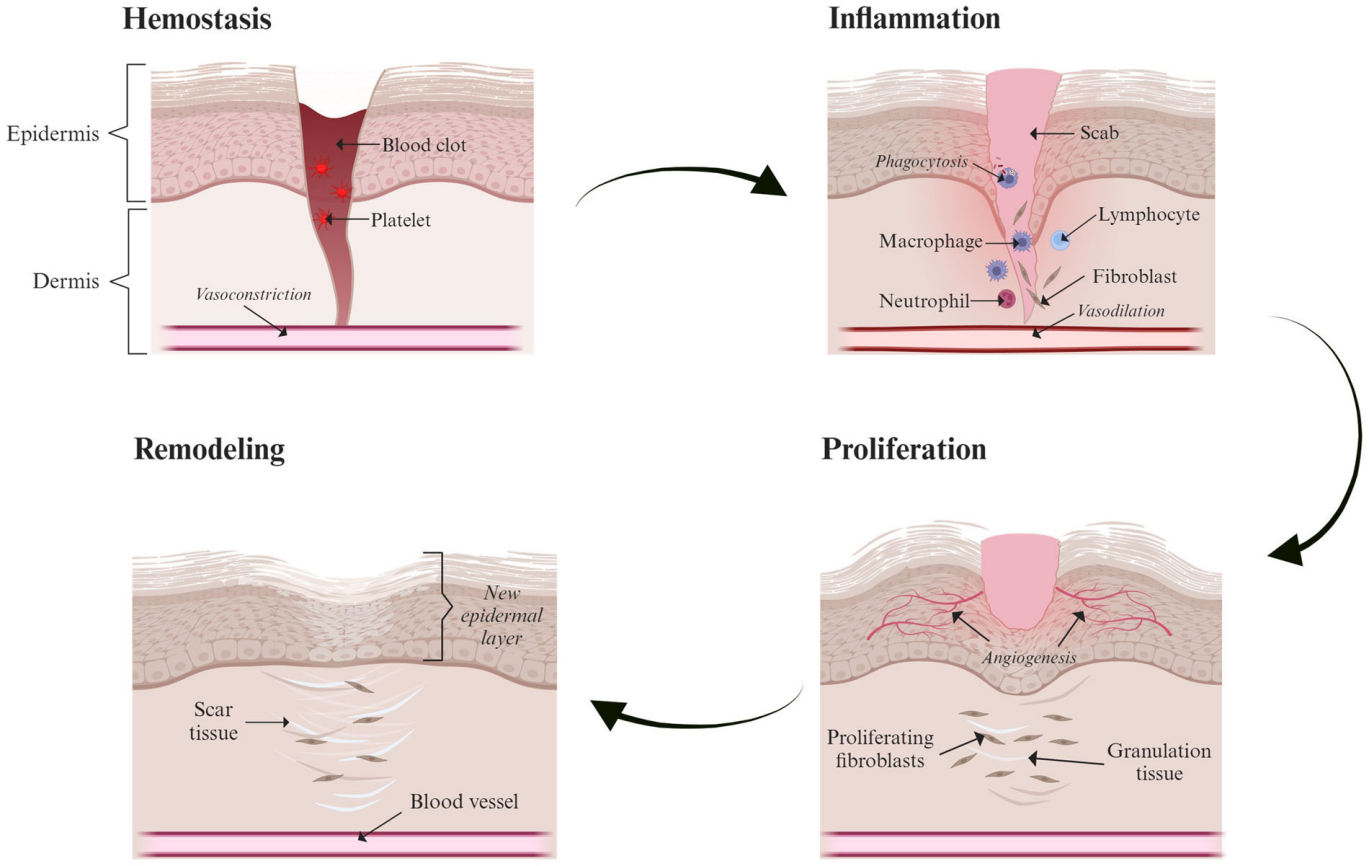
Schematic illustrations of the four stages of the normal wound healing process include hemostasis, inflammatory, proliferative, and remodeling phases. The figure was created using BioRender.com.

Wound healing is an intricate natural physiological process responding to tissue damage to prevent infection and minimize scar formation. Acute wounds self‐heal through four typical sequential phases: hemostasis, inflammation, proliferation, and tissue remodeling (Figure 1).^14^ Hemostasis involves vasoconstriction and platelets and fibrin that prevent excessing wound bleeding.^14^ Subsequently, the inflammatory phase involves vasodilation and localizing neutrophils and macrophages to clear pathogens via phagocytosis to prevent wound infection.^15^ Lymphocytes attract more macrophages to the wound area, stimulating the release of keratinocytes and fibroblasts for angiogenesis. Thereafter, the proliferative phase involves fibroblasts and endothelial cells to support capillary growth and granulation tissue production, establishing a base for the preceding wound's tissue scaffold.^15^ Lastly, the tissue remodeling phase includes collagen deposition and vascular maturation, allowing the wound to achieve maximum strength as it matures.^14^

These sequential wound‐healing phases primarily apply to acute rather than chronic wounds due to the presence of foreign bodies, bacterial biofilm colonization at the wound area, hypoxia, and secondary wound infection for the latter.^4^ Chronic wounds have an oxygen tension of 5 mmHg, much lower than the typical 20 mmHg for wound healing, making it more prone to bacterial infection.^15^ Besides, the prolonged inflammation, impaired angiogenesis, and dysregulation of the extracellular matrix (ECM) prevent wound closure, impeding the wound healing process.^16^ Comorbidities such as diabetes and obesity further complicate wound healing.^6^ The irregular shape and complex condition of chronic wounds render conventional wound dressings unsuitable for application.

## 4 TRADITIONAL AND MODERN WOUND DRESSINGS

Wound care uses mixed poultices of beer, mud, milk, herbs, and plaster containing honey, plant fibers, and animal fats for wound application.^17^ Using absorptive gauze to dry the wound site persisted for approximately two millennia.^17^ This practice continued until George Winter discovered enhanced wound healing in occluded wounds compared to exposed wounds, indicating the importance of a moist environment in the wound.^18^ Further research and clinical insights in the late 20th century regarding the significance of moist wound environments have shed light on designing modern wound dressings tailored to various wound environments. The pros and cons of traditional and modern wound dressings are summarized (Table 1).

**Table 1:** Advantages and disadvantages of traditional and modern wound dressings.

	Advantages	Disadvantages	Literatures
Traditional wound dressings			
Bandages	Economical, easy to find and use	Drug carrier only, dry, can be adhered to the wounds and cause secondary damage, require frequent replacement, and poor protection from bacterial infection.	[19, 20]
Gauzes	Economical, easy to find and use	Drug carrier only, dry, can adhere to the wound bed and cause secondary wound damage and infection, not applicable for highly exudative wounds, requires frequent replacement, and poor protection from bacterial infection.	[19, 20]
Cotton wool	Economical, easy to find and use	Drug carriers only require frequent replacement and poor protection from bacterial infection.	[19, 20]
Modern wound dressings			
Hydrocolloid	Provide a moist environment for the wounds, easy to use, integrated therapeutic substances	Can adhere to the wound and are hard to remove, unsuitable for infected wounds.	[19, 20]
Foams	Provide a moist environment for the wounds, absorbent, integrated therapeutic substances	Some require a secondary film for adherence purposes, which is not suitable for dry wounds.	[19, 20]
Hydrogels	Provide a moist environment for the wounds, is comfortable, absorbent, integrated therapeutic substances, and can be customized with a variety of polymers	Nonadherent, suitable only for minimal to moderate exudative wounds, and costly.	[19, 20]

### 4.1 Traditional wound dressings

Traditional wound dressings (bandages, gauze, and cotton wool) are easily accessible, economical, and easy to apply. However, they are generally dry and require regular dressing changes, increasing wounds' susceptibility to secondary infection and damage.^19^ Wound beds with low humidity can cause the cells to lose functionality and vitality.^17^ Conventional wound dressings are designed to be either highly or poorly adherent, where the former leads to additional pain upon removal and dressing changes, and the latter provides inadequate drainage for the wound.^9^, ^15^ For instance, applying a bandage with cotton wool tends to shed fibers that stick to the wound surface, causing pain and potential secondary bacterial infection. Additionally, some traditional wound dressings have limited absorbing capacity and oxygen supply to wounds.

### 4.2 Modern wound dressings

Modern wound dressings (hydrocolloid, hydrogel, and foam) are known for their significant moisture content, as demonstrated by the improved epithelialization of the denuded wound surfaces when moist polythene film is applied.^18^ The improved epithelialization could be due to the facile motility of keratinocytes over the moist wound site.^21^ Moisture stimulates the release of growth factors such as platelet‐derived growth factor and transforming growth factor‐beta for angiogenesis, fibrinolysis, and tissue remodeling processes in wound healing.^22^ Besides, modern wound dressings can create a comparable internal environment for the body by sustaining a relatively constant local wound temperature and humidity.^20^ Modern wound dressings can prevent microorganism invasion by forming a protective barrier between the wound bed and the external environment.^17^ Nevertheless, modern wound dressings are currently restricted to specific types of wounds, highlighting the need for further optimization for a broader range of wounds.

## 5 HYDROGEL WOUND DRESSINGS

**Figure 2:**
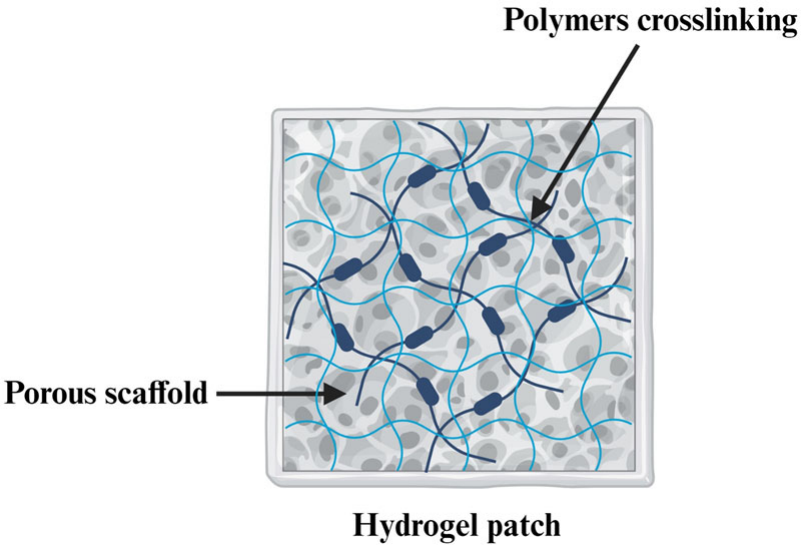
A schematic representation of a general hydrogel patch as a potential wound dressing consisting of crosslinked polymers with porous scaffold. The figure was created using BioRender.com.

The ideality of hydrogel as a wound dressing includes its high moisture content and absorption capacity, biodegradability, biocompatibility, and nonadherence.^19^ Hydrogel can be crosslinked with polymers, providing a porous scaffold for various purposes (Figure 2). Hydrogel has great hydrophilicity, conferring them a 10‐ to 1000‐fold swelling ratio of their weight equivalent, a vital aspect for absorbing wound exudates and maintaining a humid wound environment for complete epithelialization and healing.^13^, ^21^, ^23^ The moisture content and swelling capacity also help maintain hydrogel's integrity, allowing the drug solubilization and diffusion for faster wound healing.^21^, ^24^

The hydrophilicity and biodegradability of the hydrogel can alleviate pain associated with frequent dressing changes and minimize wound damage risk.^22^ Besides, hydrogels carry bioactive agents or specific drugs, endowing them with antimicrobial, anti‐inflammation, and angiogenesis abilities.^25^ Hydrogels also provide a soothing effect by decreasing the temperature of cutaneous wounds, which is especially beneficial for burn wounds.^8^ The biocompatibility of the hydrogel dressings is of utmost importance to avoid cell and antibody‐mediated rejection.^24^

## 6 BENEFITS OF HONEY TO BE INCORPORATED INTO A HYDROGEL

Honey, a natural antimicrobial agent, is again receiving attention for its potential in treating antibiotic‐resistant infections. Honey exhibits a broad spectrum of antimicrobial activity against wound pathogens such as *Staphylococcus aureus*, Methicillin‐resistant *S. aureus* (MRSA), *Pseudomonas aeruginosa*, *Escherichia coli*, and the yeast *Candida* spp.^26^ Remarkably, a 4.0%−14.8% honey concentration was reported to kill wound pathogens, including antibiotic‐resistant strains.^27^

The antimicrobial activities of honey are predominantly attributed to the peroxide and non‐peroxide activities, where the former utilizes glucose oxidase enzyme to convert glucose into gluconic acid and hydrogen peroxide via oxidation, and the latter is attributed to its high acidity (low pH).^28^ Both hydrogen peroxide and high acidity effectively kill microbes, preventing wound infection. Next, antioxidants from flavonoids and phenolic acids in honey scavenge free radicals, preventing cell damage and reducing wound inflammation.^13^ The high saccharide content (30% glucose, 40% fructose, 5% sucrose, and small amounts of disaccharides) accounts for high honey viscosity that provides a protective barrier between wound beds and dressings, preventing bacterial growth.^13^, ^29^ The honey's water content further increases the hydrogel's moisture, preventing wound dehydration and additional pain upon dressing application. To sum up, honey's hydrophilicity, high osmotic pressure, and viscosity have contributed to excellent moisture, reducing pain and wound exudates, which were demonstrated in several studies (Table 2).^32^ The honey's original fragrance was also postulated to remove the wounds' malodor.^28^

**Table 2:** Previous honey applications in wound dressings.

Wound dressing	Targets	Methodologies	Results	References
Medihoney^TM^ (A honey‐colloid dressing with a proprietary blend of honey applied to a sterile dressing pad with approximately 20 g of honey per application)	40 patients with non‐healing leg ulcers	It was a 12‐week study. The wounds were assessed using Doppler ultrasound and measured through digital imaging, subsequently evaluated fortnightly. Questionnaires were employed to gauge their self‐reported pain levels and the presence of odor after Medihoney treatment.	50% of the patients experienced a reduction in reported pain levels. Among the 26 patients with malodorous wounds, all experienced a significant decrease in the average overall odor level, with 11 patients noting the absence of any remaining odor. Additionally, there was a noteworthy reduction in the size of the wounds in 20 patients' ulcers.	[30]
Medihoney^TM^ (Medihoney Antibacterial Wound Gel)	8 patients with surgical leg wounds	The wound progress was monitored over 1−2 months by measuring the wound size and self‐evaluating the pain, exudate, and malodor levels.	The results showed a significant reduction in wound sizes, with some achieving complete healing. The surgical wounds had a notable decrease in odor, pain, and exudate. Almost all patients exhibited 100% wound granulation or epithelialization, indicating nearly complete healing of the wounds.	[31]

Aside from the above‐mentioned, honey also has various mechanisms of action in healing wounds (Table 3). Honey prevents wound infection by disrupting the quorum‐sensing systems that regulate biofilm formation.^34^ Honey's high sugar concentration and osmolarity create a hypertonic environment that draws the water content out from the bacterial cells, killing the bacteria. Both bee defensin‐1 peptide and methylglyoxal (MGO) impair biofilm formation by perforating bacterial membranes, inhibiting nucleic acids and protein synthesis, and preventing bacterial adhesion to surfaces, respectively.^43^ In contrast, hydrogen peroxide kills bacteria via oxidative stress, causing irreversible DNA damage.^43^ Honey aids in reducing inflammation from edema and allows the diffusion of oxygen and nutrients in the microvasculature of wound tissue for wound repair.^38^ The reduction of reactive oxygen species and reactive nitrogen species by honey can prevent the occurrence of hypergranulation and fibrosis that potentially lead to hypertrophic scarring.^38^ The constituents of honey have also been speculated to promote proangiogenic action, providing moisture and a local cellular energy source for endothelial cell proliferation and healthy tissue granulation.^38^ The polyphenol compounds (flavonoids and phenolic acids) have also contributed to the immunomodulatory action of honey.

**Table 3:** Mechanisms of action (MOA) of honey contributing to wound healing and the related studies.

MOA	Descriptions of honey MOA	Related studies
Antimicrobial	QS and microbial biofilm disruption, leading to the loss of microbial coordinated responses, fail to secrete virulence factors and eventually become harmless and defenseless.^33^ Honey inhibits bacterial cell adhesion to surfaces, suppressing their metabolic activities, and downregulating the global QS regulator genes, eventually preventing biofilm development.^34^	A 2011 study by Lee et al. demonstrated that honey concentration at as low as 0.5% (v/v) could reduce the biofilm formation of *Escherichia coli*.^35^ Their transcriptomic study also revealed that honey can inhibit biofilm‐related curli genes, QS genes, and virulence genes in *E. coli*, mainly attributed to the glucose and fructose in the honey.^35^ A 2014 study by Lu et al. showed that Manuka honey can inhibit biofilm formation due to the diffusion of MGO compound into the biofilm matrix and acts by killing the bacteria.^36^ A 2021 study by Proaño et al. showed that the synergistic effects of the high sugar concentration, the presence of hydrogen peroxide, and bee defensin‐1 peptide in honey suppress the biofilm formation.^37^
Anti‐inflammatory	Honey reduces prostaglandin levels in the plasma by impeding the activity of COX1 and COX2; attenuating the NF‐κB, inhibiting the expression of TNF‐α and NO, eventually reducing inflammation.^38^	N/A
Antioxidant	The flavonoids and phenolics of the honey inhibit the superoxide anions‐producing enzymes and thus reduce the production of ROS and RNS.^38^	N/A
Proangiogenic	Honey stimulates angiogenesis, an essential part of the proliferative phase of wound healing.	A 2010 in vitro angiogenesis analogues study by Rossiter et al. showed that honey exerted its proangiogenic effects at as low as 0.1%−1.0% (v/v).^39^ They showed that honey was as strong a stimulator of pseudotubule formation as VEGF in the rat aortic ring assay, indicating the potency of honey to stimulate blood vessel formation.^39^ A 2020 in vivo study by Chaudhary et al. demonstrated significant wound closure, reepithelialization, and collagen deposition in the diabetic mice model upon application of 0.1% (v/v) Jamun honey.^40^
Immunomodulation	Honey promotes the release or activation of immune system mediators, stimulates the mitogenesis of B and T lymphocytes, and activates neutrophils and macrophages to remove potential infection‐causing pathogens.^38^ The level of serum antibodies (IgM, IgG, and IgA) are augmented by honey to eliminate the infection‐causing organisms.^41^	A 2007 study by Tonks et al. showed that honey can modulate the TLR4 pathway by inducing the production of TNF‐α and IL‐1β, which in turn stimulates the release of PDGF, which is crucial in fibroblast development for tissue repair.^42^

Abbreviations: COX1 and 2, cyclooxygenases 1 and 2; IL‐1β, interleukin‐1β; MGO, methylglyoxal; N/A, non‐applicable; NF‐κB, nuclear factor kappa B; NO, nitric oxide; PDGF, platelet‐derived growth factor; QS, quorum sensing; RNS, reactive nitrogen species; ROS, reactive oxygen species; TLR4, toll‐like receptors 4; TNF‐α, tumor necrosis factor‐alpha; VEGF, vascular endothelial growth factor.

Nonetheless, direct application of honey in its liquid form on wounds like burns would be a nuisance as it may further liquefy as temperature increases, leading to honey leakage from the wound areas.^44^ In high‐exuding wounds, honey could be diluted, resulting in limited therapeutic effects.^45^ Thus, honey is suspended in the hydrogel to improve these limitations.

## 7 THE NEED TO INCORPORATE OTHER POLYMERS INTO THE HONEY HYDROGEL

Honey decreases the mechanical strength of hydrogel, as demonstrated by a decrease in gellan‐gum film tensile strength with high honey concentration ([2% vs. 10% [w/v] honey at 2.1 vs. 0.9 MPa, respectively]).^46^ Besides, honey may entrap within the honey hydrogel, restricting the honey's therapeutic effect on the skin's superficial layers, causing further delay in healing deep wounds such as burns and crush injuries.^47^ Henceforth, other polymers (natural or synthetic) are required to create an optimum hydrogel dressing for different wound types. The notable examples of natural (CS and gelatin) and synthetic polymers (PVA and PEG) will be further discussed concerning the properties such as hydrophilicity, biocompatibility, biodegradability, and structural integrity, to name a few.

### 7.1 Chitosan

Chitosan is a natural polycationic polysaccharide derived from the *N*‐deacetylation of chitin, the skeletal material of crustaceans, fungi, and insects.^48^ Akin to honey, CS has antimicrobial properties that can be activated by the protonation of CS's amine groups ([−NH_2_] to [−NH_3_^+^]) in an acidic medium, permeabilizing the microbial negatively‐charged cell membranes, releasing intracellular components.^49^, ^50^, ^51^, ^52^ CS can adhere to negatively charged surfaces, including the skin's stratum corneum.^53^, ^54^ CS's hemostatic nature was demonstrated by the shortened hemostasis time in rat models compared to conventional gauze dressing.^55^ A retrospective study showed a significant improvement in wound healing, degree of pain, and wound itchiness in 80 patients with chronic refractory wounds treated with CS‐based hydrocolloid dressing for 3 weeks.^56^ CS shortens the inflammatory phase by facilitating the migration of macrophages and other active cells to clear the inflammatory mediators and cellular debris at the wound site, improving wound healing.^57^ CS‐based oligosaccharides significantly reduce lipopolysaccharide‐induced release of proinflammatory cytokines such as IL‐1β, IL‐6, and TNF‐α from the THP‐1 monocytes, indicating the anti‐inflammatory potential of CS.^58^ In addition, CS contains glucosamine and *N*‐acetylglucosamine, where the latter is structurally similar to hyaluronic acid (HA), an ECM macromolecule that favors natural HA synthesis, activating extrinsic clotting pathways, promoting keratinocyte migration and proliferation, and enhancing reepithelialization.^57^, ^59^ The structural resemblance of CS to HA confers excellent biocompatibility of CS hydrogel to promote wound healing. CS hydrogel has promising safety where 70% cell viability was observed on normal human dermal fibroblasts.^60^

CS hydrogel has a relatively rapid biodegradation rate in different wound‐healing moments. In an exudative wound environment with pH 8.5 and 9, CS hydrogel showed a greater biodegradation rate with mass loss of 42% and 45%, respectively, than the physiological condition (pH 7.4) at only 32% over a 14‐day setting.^60^ The greater biodegradation rate of the hydrogel allows for a faster release of bioactive compounds to tackle infection and support exudative wound repair. At normal dermis conditions with pH 5.5, CS‐based hydrogel biodegrades rapidly with a mass loss of 75% on the first day and completely degraded on the second day, indicating the ability of the hydrogel to be completely absorbed into the newly generated tissue, favoring smooth tissue regeneration without the need for traumatic debridement.^60^ Moreover, the hydrogen bonding between the amine and hydroxyl groups of the CS alone can act as a gelling polymer, contributing to the mechanical strength of the hydrogel.^57^

Interestingly, CS alone was reported to lack significant antimicrobial activity against the common wound pathogens, *P. aeruginosa* and *Candida albicans*.^61^ Instead, CS/honey hydrogel, with the optimal ratio of 1 CS: 3 honey, effectively inhibited pathogens' growth compared to CS or honey alone.^61^ The addition of intact comb waxy honey in the CS hydrogel has resulted in a remarkably low minimum inhibitory concentration (MIC) at 0.0625% and 0.03125% for *P. aeruginosa* and *C. albicans*, respectively.^61^ Upon combining CS and honey, the MIC of honey against wound pathogens also significantly decreased, illustrating a synergistic antimicrobial effect.^61^ The honey's acidic property might cause the synergistic antimicrobial property to protonate CS, activating its polycationic properties to tackle wound pathogens.

### 7.2 Gelatin

Gelatin is a natural polypeptide derived from the hydrolysis of non‐soluble collagen in animal cartilage, bones, and skins.^62^ The proline, glycine, and hydroxyproline compositions in gelatin mimic the ECM, providing excellent biocompatibility and thus suitable for wound healing applications.^63^, ^64^ Gelatin stimulates hemostasis by promoting thrombus development, and its glycine composition further promotes platelet attachment to form blood clots in the wound area.^63^ The porosity of the gelatin scaffold then allows sufficient nutrients and oxygen permeability to the cells, in addition to fibroblast migration to the wound site, facilitating new tissue formation and angiogenesis.^62^, ^63^ The hydrophilicity of gelatin retains moisture on wounds and allows for rapid drug release by spreading the water content out from within the gelatin.^63^

However, the water‐soluble nature renders gelatin poor mechanical strength with a rapid biodegradation rate, making it undesirable as a base material in hydrogel for wound healing.^63^ Poor mechanical strength and rapid biodegradation rate of gelatin can cause hydrogel dressing instability and the failure of honey‐sustaining release (as a bioactive compound) to the wounds. Gelatin undergoes a reversible sol‐gel transformation through controlled cooling, with the temperature maintained below its melting point. When placed in cold water, gelatin absorbs water 5−10 times its mass and swells.^62^ The stability of gelatin triple helices increases with the increasing content of proline and hydroxyproline amino acids, subsequently increasing its thermal stability.^62^ Nonetheless, gelatin as a protein will still be denatured upon long‐term exposure to temperatures above 40°C.^62^ Hence, the swelling ability of gelatin renders it suitable for wound dressings, except for burn wounds, due to its thermal instability. Despite the benefits of gelatin as a biopolymer, it has limited antimicrobial efficacy. Encapsulating another polymer with superior antimicrobial properties, such as honey, can improve this.^63^

The absence of a well‐established study on gelatin/honey hydrogel could be attributed to both polymers' inadequate mechanical strength and water‐soluble characteristics. These properties hinder their ability to independently form a stable, higher endurance hydrogel with sustained degradation. These limitations have since then been addressed with the crosslinking of gelatin with synthetic polymers (PVA or PEG) or other natural polymers (CS).^65^, ^66^, ^67^

### 7.3 Poly(vinyl alcohol)

Poly(vinyl alcohol) is a synthetic long‐chain polymer derived from poly(vinyl acetate) by alcoholysis, hydrolysis, or ammonolysis.^68^ PVA has garnered sufficient attention due to its biocompatibility, high chemical resistance, significant mechanical strength, low toxicity, and excellent biodegradability.^69^, ^70^ The biocompatibility of PVA extends to its relatively safe and nontoxic properties to fibroblast cells, retaining 80% cell viability post‐72 hours of treatment.^71^ Its biocompatibility extends further, where it can remain in contact with body tissues for prolonged periods without interacting with the tissues and inducing detrimental immune reactions to the host, exhibiting promising bio‐inert levels. In particular, this was demonstrated with fewer to no immune cells (macrophages and giant cells) around the PVA hydrogel particles after 3 months of in vivo tests.^72^ Its bio‐inertness is speculated to be an attribution of PVA for not forming any bonds with the body tissues and, thus, can be chemically or topographically modified.^73^ The semicrystalline structure of PVA hydrogel gives cells good oxygen and nutrient permeability, which enhances wound healing by promoting the growth of new tissues.^69^ Although the exact role of PVA in bleeding control has yet to be unraveled, the application of PVA hydrogel was shown to significantly reduce the bleeding time (187−108 s), bleeding volume (2.98−1.84 mL) and increased survival (75%−91.7%) in rats.^71^ PVA can create crosslinking points within crystalline clusters via repeated freeze‐thaw cycles, exhibiting excellent gel‐forming characteristics that can crosslink with other polymers and increase the mechanical strength of a hydrogel.^71^ PVA's hydrophilicity, although weaker than that in natural polymers, can still maintain moisture and absorb exudates from the wound.^74^ Nevertheless, the lack of PVA's biological activity confers limited antimicrobial and cell growth‐promoting function, requiring the supplement of additional beneficial composite polymers for hydrogel wound dressing.

### 7.4 Polyethylene glycol

Polyethylene glycol, a petroleum‐based synthetic polymer composed of polyether compounds, is widely used in many fields, from industrial manufacturing to medicine.^75^ It has caught much attention due to its biocompatibility, biodegradability, and hydrophilicity, to name a few.^70^ Naturally, PEG is bioinert with no intrinsic biological activity and low immunogenicity as the cells cannot attach to PEG.^76^ However, PEG can be fabricated to exhibit antimicrobial activity where modified PEG has *E. coli* and *S. aureus* inhibited at an efficiency of 64.1% and 93.5%, respectively.^76^, ^77^ PEG can also be modified to include cell‐promoting characteristics for cell proliferation and migration.^77^ Additionally, PEG does not affect the drug's efficacy in the hydrogel, as demonstrated by the larger agar inhibition zone loaded with PEG and antibiotics than by PEG alone.^77^ This further affirms the possibility of honey/PEG for enhanced antimicrobial effect in the hydrogel. PEGs are prepared by the polymerization of ethylene oxide, which can be made in a wide range of molecular weights that are either linear or branched with hydroxyl groups (−OH) as the end groups.^78^ The increased hydroxyl groups and molecular weight in PEG have enhanced its antimicrobial activity by out‐competing the bacteria for water.^79^ PEG hydrogel has relatively uniform porous networks, making it efficient for nutrient transportation in wound dressings.^77^ Additionally, PEG hydrogel has excellent gradual degradability, exceeding 50% degradation over 21 days, avoiding frequent dress changing.^77^ The absence of cell lysis and cell growth reduction upon coculturing with PEG hydrogel also showed outstanding biocompatibility of PEG hydrogel.^77^ More importantly, the FDA has approved PEG due to its biodegradability, rapid excretion from living organisms, and minimal toxicity.^80^ The PEG's conformational flexibility and excellent chain mobility allow them to conjugate with other polymers, which can be incorporated in the hydrogel dressing with various polymers, contributing to increased mechanical strength.^81^, ^82^

However, incorporating PEG into a hydrogel of other polymers can alter its properties. This was observed in a study where PEG reduces gelatin hydrogel's tensile strength and hydrophilicity. A study showed that 15% (w/v) of PEG in gelatin hydrogel confers optimum mechanical properties.^82^ Hence, further studies are required to investigate the optimal ratio of PEG and other polymers in a hydrogel to achieve maximum efficacy in wound healing.

### 7.5 Honey‐based hybrid hydrogels

The application of honey‐based hybrid hydrogels, incorporating two or more polymers, has captured sufficient attraction, and it was investigated in several studies (Table 4). The hydrogels are mainly composed of the combination of honey/CS/gelatin and honey/CS/PVA, while there are limited studies on honey/PEG hydrogel; instead, crosslinking with the other non‐discussed polymers, such as cellulose and polyvinyl pyrrolidone, was explored.^32^, ^89^ Nonetheless, employing honey as a drug in PEG hydrogel is possible as its porous structure can act as a drug carrier that transports honey to the wound without affecting its efficacy. Its hydrophilicity can also keep the moisture around the wound and absorb wound exudates. Further studies can delve into the combination of honey/PEG with the discussed polymers (CS, gelatin, or PVA) to investigate the pros and cons among them.

**Table 4:** Summaries of the findings on honey‐based hybrid hydrogels.

Honey‐based hybrid hydrogels	Findings	References
Honey/CS/gelatin HS	The HS containing honey swelled and reached equilibrium within 5 min compared to those without honey (30 min). CS and honey exert synergistic antimicrobial activity against *Staphylococcus aureus* and *Escherichia coli* (100% inhibition rate even after 3 days). HS demonstrated complete healing in burn wounds for approximately 12 days, compared to 14 days with commercial burn ointment (MEBO® ointment). Honey accelerates HS degradation under high temperatures (burn wounds), dispersing honey into the water. Honey reduces the stiffness of HS. With 20% honey, HS was found to mimic the mechanical behavior of natural skin, providing a desirable tissue repair environment.	[83]
Honey/CS/gelatin hydrogel	As gelatin mass increased from 10 to 20 g, the crosslinking between CS and gelatin reduced from 68.86% to 65.68%, respectively, attributed to the reduced polycationic site exposure of CS, restraining ionic bond formation between CS's cationic ammonia and gelatin's carboxylate groups, reducing the hydrogel's mechanical strength. Honey further reduced gel fraction by disturbing the ionic interaction between CS and gelatin. Honey interfered with the hydrogen bond formation between water molecules and the crosslinked CS/gelatin network, reducing the swelling index. CS increased the swelling index of the hydrogel from 300% to 400%, likely due to the increased amine (‐NH‐) and hydroxyl (−OH) groups of CS, hastening hydrogen bond interaction with water, thereby improving water absorption capability. Instead of honey, CS was reported to be responsible for the hydrogel's antimicrobial activity toward *E. coli* and mold, aligning with another study.^84^	[85]
Honey/CS/PVA hydrogel	The crosslinking of PVA with CS promoted greater swelling capacity than that of pure PVA hydrogel due to the increased hydrophilic functional groups (−OH and ‐NH‐) contributed by CS. Adding CS into PVA hydrogel, which is naturally non‐antimicrobial, was also shown to increase the antibacterial activity where *E. coli* grows on plates with PVA hydrogel but not with CS.	[86]
Honey/CS/PVA DN‐Hyd	CS acts as the first polyelectrolyte gel network and PVA as the second neutral network, while honey acts as a modifier for its excellent biodegradability and biocompatibility. CS increases the mechanical and tensile strength and elongation at break values, enhancing the hydrogel's stretchability for wounds with various sizes. Honey was previously shown to expedite PVA hydrogel's thermal degradation due to its low thermal stability and reduce the hydrogel's water content (swelling behavior), but these were mitigated by CS inclusion. The biocompatibility of the DN‐Hyd system was confirmed with the absence of altered cell morphology in treated cells.	[87]
Honey/CS/PVA hydrogel	The tensile strength of the hydrogel resembles average human skin at 21.6 ± 8.4 MPa, a crucial property of wound dressing that mimics naturally healthy skin, preventing hydrogel tearing upon stretching. However, the presence of honey can also reduce the tensile strength and increase elasticity due to the possible role of honey as a plasticizer in PVA/CS hydrogel. The sustained biocompatibility of the hydrogel was excellent at above 70% cell viability in vitro post‐30‐day incubation, possibly attributed to the saccharides of honey as nutrients for the cells. The PVA/CS/honey hydrogel revealed a staggering 98% wound closure compared to the control group at 89%, indicating its remarkable effectiveness in treating wounds.	[50]
Honey/CS/PVA hydrogel	The hydrogel's moisture content and swelling ratio increased with increasing CS concentration. The hydrogel has higher mechanical properties than pure PVA or CS hydrogel due to the complementary strong crosslinking interactions between CS and PVA, rendering them a controllable drug delivery system via sustained release of honey (drug) from the hydrogel. CS reduced WVTR which is crucial in wound healing by retaining moisture within and between hydrogel and wound beds. The hydrogel exhibited synergistic antimicrobial properties against *S. aureus* (5.01 ± 0.32 mm diameter of ZOI).	[88]

Abbreviations: CS, chitosan; DN‐Hyd, double network‐hydrogel; HS, hydrogel sheet; PVA, poly(vinyl alcohol); WVTR, water vapor transmission rate; ZOI, zone of inhibition.

**Figure 3:**
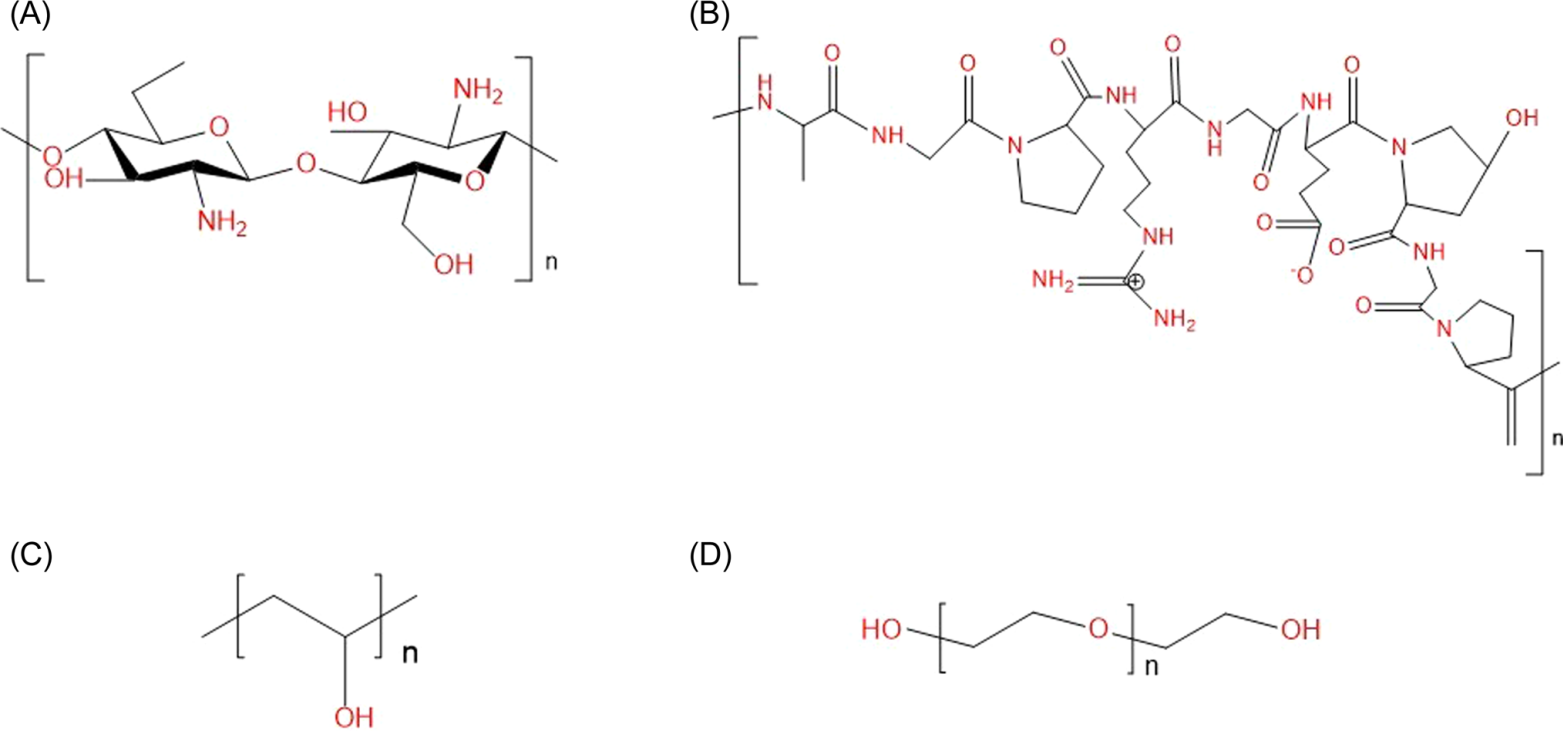
Chemical structures of (A) CS, (B) gelatin, (C) PVA, and (D) PEG. The hydrophilic functional groups (‐C‐O‐C‐, ‐OH, ‐NH_2_, ‐NH‐, ‐C=O, and ‐C‐N‐C‐) were colored in red. CS, chitosan; PEG, polyethylene glycol; PVA, polyvinyl alcohol.

The pros and cons of hydrogel with natural, synthetic, and hybrid polymers were summarized (Table 5). To sum up, natural polymers contribute to biological functions. In contrast, synthetic polymers are more concerned with the mechanical strength and elasticity of the hydrogel, allowing the sustained release of honey (drug) in the hydrogel. More natural polymers can be added to enhance the swelling behavior of the hydrogel as they have more hydrophilic groups than synthetic polymers (Figure 3), allowing greater water absorption capacity.^90^ The swelling capacity of hydrogel requires adjustment as excessive swelling can impede wound healing by causing a mismatch between the hydrogel and the wound morphologies and the fluid imbalance of the ECM.^91^ This can lead to increased pressure on the surrounding tissue, impairing blood flow, and potentially cause pain and discomfort in the wound area. Conversely, insufficient swelling hampers the absorption of wound exudates, nutrients, and oxygen, increasing the risk of infection and delaying wound healing.^13^

**Table 5:** The advantages, disadvantages, and implications of utilizing natural, synthetic, and hybrid polymers in a hydrogel for wound healing.

	Advantages	Disadvantage(s)	Implication(s)	References
Natural polymers				
CS	Hemostatic property Pain reduction Shortened inflammatory phase Biocompatible It can act as a gelling polymer	No significant antimicrobial activity when used alone	Other polymers (such as honey) are required to exhibit substantial antimicrobial properties.	[50, 61, 85]
Gelatin	Biocompatible Enhance hemostasis Promote angiogenesis Can retain moisture on wounds Allows rapid drug release	Poor mechanical strength Rapid biodegradation rate Thermal instability Absence of antimicrobial effects	Other polymers (such as CS and synthetic polymers) are required to improve the limitations stated.	[62, 63]
Synthetic polymers				
PVA	Biocompatible Good mechanical strength Low toxicity Biodegradable Bioinert Allows good oxygen and nutrient permeability to cells Hydrophilic	Lack of antimicrobial and cell growth promotion properties	Bioactive and antimicrobial natural polymers (such as honey and CS) are required.	[69, 71]
PEG	Hydrophilic Biocompatible Biodegradable Allow efficient nutrient transportation Minimal toxicity Bioinert Good mechanical strength	Its incorporation can alter the properties of the hydrogel, which consists of other polymers Lack of antimicrobial properties naturally	Bioactive and antimicrobial natural polymers (such as honey and CS) are required.	[76, 77]
Hybrid polymer				
Honey/CS/gelatin	Rapid and greater exudate absorption Non‐cytotoxic Synergistic antimicrobial activity by CS and honey	Honey weakens hydrogel's mechanical strength	The ratio of each component in the hydrogel should be studied and optimized to achieve a desirable hydrogel for wound healing.	[83, 85]
Honey/CS/PVA	Improved mechanical strength, thermal stability, and moisture content properties Reduction in WVTR Biocompatible Similar tensile strength to human's skin Antimicrobial	Honey weakens hydrogel's mechanical strength	The ratio of each component in the hydrogel should be studied and optimized to achieve a desirable hydrogel for wound healing.	[50, 87, 88]

Abbreviations: CS, chitosan; PEG, polyethylene glycol; PVA, poly(vinyl alcohol); WVTR, water vapor transmission rate.

Besides, the biodegradation rate requires adjustment as it can affect cell growth and new tissue regeneration on the wound site.^76^ Synthetic polymers generally have higher mechanical strength but lower biodegradation rates than natural polymers (Table 6). Hence, a hybrid of synthetic and natural polymers allows hydrogel fabrication with optimal mechanical and biodegradation properties, permitting a controllable and sustained drug delivery system. This could be observed when the gelatin/CS/honey hydrogel showed a non‐synergistic antimicrobial effect of CS and honey, which might be ascribed to the weak hydrogel matrix for simultaneous honey and CS delivery.^85^ However, the more robust PVA/CS/honey matrix allows more stable delivery of CS and honey, resulting in a synergistic antimicrobial effect.^88^

**Table 6:** Comparisons between the general features of natural and synthetic polymers and their main potential improvement when incorporated into honey hydrogel.

Properties	Natural	Synthetic	Hybrid	Literature
Polymers	CS, gelatin	PVA, PEG	Honey/CS/gelatin, PVA/CS, honey/CS/PVA	N/A
Cytotoxicity	Non‐cytotoxic	Non‐cytotoxic	Non‐cytotoxic	[69, 83]
Biocompatibility	Yes	Yes	Yes	[63, 69]
Natural bioactivity	Bioactive	Bioinert	Bioactive due to the presence of natural polymer(s)	[63, 69]
Mechanical strength	Poor	Good	Good	[63, 69]
Swelling behavior	Good	Poor	Good	[63, 69]
Biodegradation rate	Fast	Slow	Moderate	[63, 92]
The main potential improvement of honey hydrogel upon crosslinking with the polymers	Enhanced swelling behavior	Enhanced mechanical property	Enhanced swelling and mechanical strength, optimized biodegradation rate	[83, 88]

Abbreviations: CS, chitosan; N/A, not applicable; PEG, polyethylene glycol; PVA, poly(vinyl alcohol).

The suitable mechanical strength of the hydrogel is crucial to ensure that the hydrogel is elastic enough to be stretched and yet not too brittle to rupture easily.^93^ Hydrogel that is too strong will cause discomfort to the patients due to the restriction of mobility.^94^

## 8 PROSPECTS OF HONEY HYDROGEL WOUND DRESSING

Despite the potential benefits of incorporating various polymers into the honey hydrogel, more comprehensive research is needed on its practical applications in wound therapy. Hence, other aspects should be considered in the future for fabricating a user‐friendly and practical honey hydrogel dressing for various wounds (Table 7).

**Table 7:** Aspects to be considered in fabricating a feasible honey hydrogel wound dressing.

Aspects	Outcomes
The type, dilution, and concentration of honey	The healing efficacy of hydrogel in different wound applications
The ratio of polymers and honey	A desirable dressing for various wounds
The hydrogel's thickness and porosity	A desirable dressing for wounds with varying severity and depths
The hydrogel's thermal degradation	A desirable dressing for various wounds, especially burn wounds
The production process, conditions, and cost concerning honey hydrogel	An affordable and commercialized hydrogel dressing
Exploring other polymers besides those discussed	A honey hydrogel with enhanced wound‐healing properties via various combinations of polymers
Exploring mixtures of synthetic and natural materials for hydrogel^95^	An enhanced hydrogel wound dressing that can respond to changes from harsh external environments^95^

## 9 CONCLUSION

**Figure 4:**
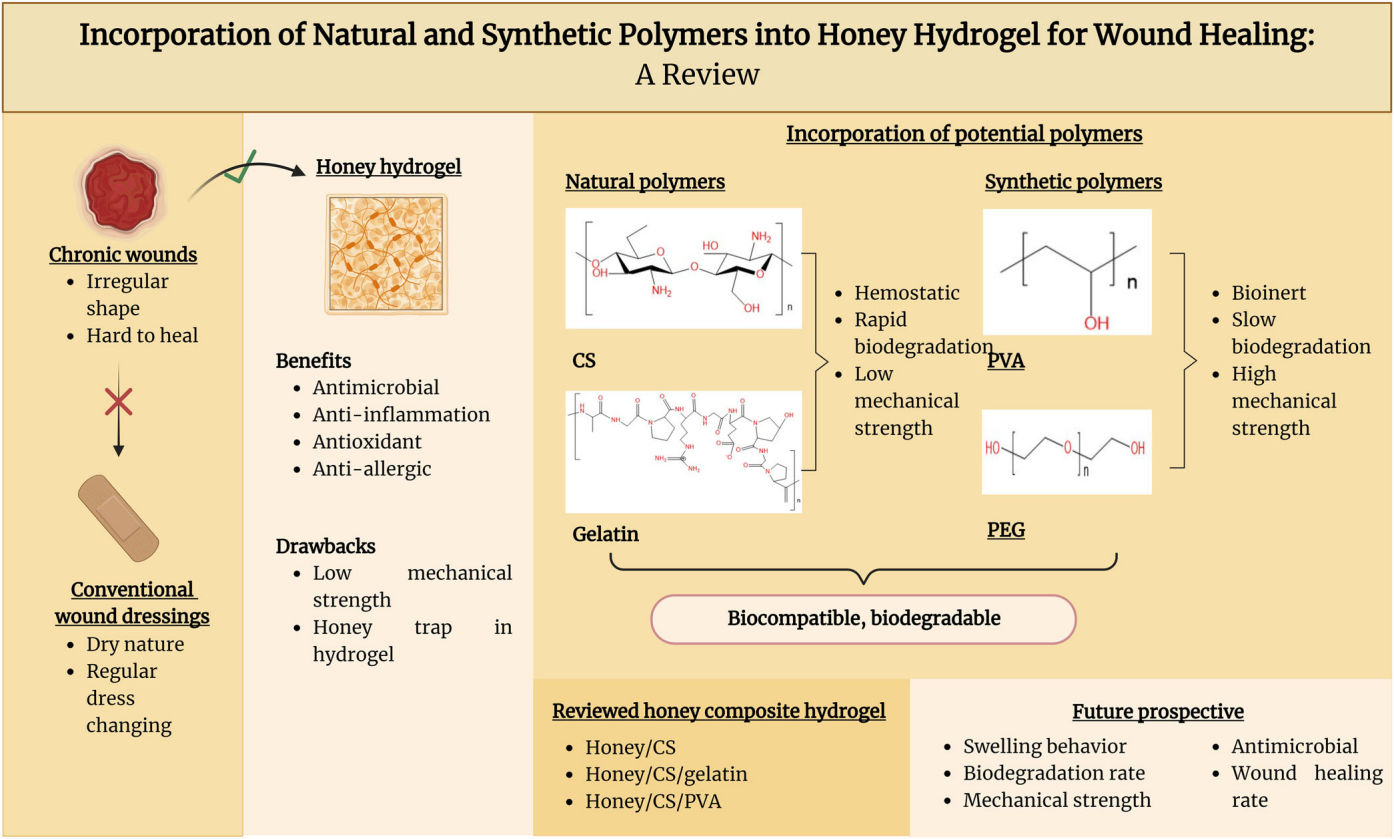
Summary of the role of honey hydrogel and the incorporation of selected polymers into the honey composite hydrogel. The figure was created using BioRender.com.

The enormous economic burden and difficulty in managing chronic wounds necessitate modern dressings. The honey hydrogel was the protagonist in this review due to the lack of in‐depth studies on honey hydrogel and its beneficial roles, including antimicrobial, anti‐inflammatory, antioxidant, anti‐allergic, and proangiogenic properties.^96^ However, honey's incapability to stand alone in hydrogel prompts exploring the possible polymers to be incorporated into the hydrogel. Given this, the incorporation of natural (CS and gelatin) and synthetic (PVA and PEG) polymers was explored and compared (Figure 4). With the information garnered from this review, it is hoped that the fabrication and commercialization of the desired honey hydrogel for wound treatment could be brought forth.

## AUTHOR CONTRIBUTIONS

Siau Wui Chin prepared the manuscript. Adzzie‐Shazleen Azman and Ji Wei Tan conceived the idea, reviewed the drafts, and provided important information for the completion of the manuscript. All authors approved the submission of the final version of this manuscript. All authors have read and approved the final version of the manuscript.

## CONFLICT OF INTEREST STATEMENT

The authors declare no conflict of interest.

## TRANSPARENCY STATEMENT

The lead author, Ji Wei Tan affirms that this manuscript is an honest, accurate, and transparent account of the study being reported, that no important aspects of the study have been omitted, and that any discrepancies from the study as planned, (and, if relevant, registered) have been explained.

## Data Availability

Data sharing is available upon request. Ji Wei Tan had full access to all of the data in this study and takes complete responsibility for the integrity of the data and the accuracy of the data analysis.
